# Pros and cons of reporting incidental findings in lung cancer screening

**DOI:** 10.1007/s00330-025-11580-7

**Published:** 2025-04-15

**Authors:** Roberta Eufrasia Ledda, Gianluca Milanese, Marie-Pierre Revel, Annemiek Snoeckx

**Affiliations:** 1 Department of Medicine and Surgery (DiMeC), University of Parma, Parma, Italy; 2 Thoracic Surgery Unit, Fondazione IRCCS Istituto Nazionale Tumori, Milan, Italy; 3 Department of Radiology, Hôpital Cochin, Assistance Publique-Hôpitaux de Paris, Paris, France; 4 Université Paris Cité, Faculté de Médecine, Paris, France; 5 Antwerp University Hospital, Edegem, Belgium; 6 Faculty of Medicine and Health Sciences, University of Antwerp, Wilrijk, Belgium

**Keywords:** Lung cancer screening, Low-dose computed tomography, Incidental findings

## Abstract

**Abstract:**

Incidental findings (IFs) are common in lung cancer screening (LCS). While the detection of some of these findings can lead to early diagnosis and treatment of clinically significant conditions, it also carries the risks of overdiagnosis and overtreatment, causing anxiety for patients and increased economic costs for health systems. Effective management of IFs requires a balanced approach guided by clear guidelines, standardized reporting, and participants-centered communication. As the field of LCS evolves, continued research and innovation will be essential in refining the strategies for managing IFs, ensuring that the benefits of screening are maximized while minimizing potential harm. Evidence-based guidelines on reporting and management of IFs, however, are still lacking. This narrative review explores the pros and cons of reporting IFs in LCS, focusing on key controversies.

**Key Points:**

- *Reporting and managing incidental findings in lung cancer screening is largely debated*.
- *The detection of incidental findings can lead to early diagnosis of clinically significant conditions but carries the risks of overdiagnosis and overtreatment*.
- *A balance must be found to have a positive impact on the population while not placing a burden on healthcare systems*.

## Background

Based on the evidence of a significant reduction in lung cancer (LC)-related mortality in high-risk individuals [1, 2], low-dose Computed Tomography (LDCT) lung cancer screening (LCS) is increasingly endorsed by national stakeholders and scientific societies [3, 4]. On December 9, 2022, the Council of the European Union modified its recommendations concerning LCS, proposing countries to explore the feasibility and effectiveness of screening with LDCT [5]. With the expansion of LCS programs, addressing the well-recognized issues related to reporting and managing incidental findings (IFs) has become crucial. IFs are defined as abnormalities detected on LDCT that are outside the scope of LCS [6]. These findings are common, with some studies reporting that more than half of the individuals undergoing LDCT-LCS present with at least one IF. Notably, LCS participants, though considered “healthy,” often have comorbidities, most commonly chronic pulmonary disease, coronary cardiovascular disease, and diabetes [7].

IFs can be categorized into thoracic (intrapulmonary and extrapulmonary) and extrathoracic abnormalities. While extrathoracic abnormalities are generally accepted as “incidental,” some thoracic ones are considered not strictly incidental but rather a reflection of comorbidities. These include pulmonary emphysema, coronary artery calcification (CAC) and smoking-related interstitial abnormalities [8, 9]. Some authors have suggested using “additional” instead of “incidental” for those findings beyond pulmonary nodules [10]. Another proposed classification distinguishes clinically significant from insignificant findings [11]. Regarding the former, providing an exhaustive list is not possible, but they include major aortic dilatation, massive pleural effusion, and mediastinal bulky lymphadenopathies, requiring further diagnostic steps.

The detection of IFs in LCS is a double-edged sword. Identifying abnormalities offers an opportunity for early detection of serious conditions, with the potential of improving overall outcomes. However, LDCT protocols might not adequately characterize most of these abnormalities, requiring additional imaging tests. This can lead to a cascade of additional tests, procedures, and consultations, some of which may be unnecessary or even potentially harmful [12].

The reporting and management of IFs remain debated. Evidence-based guidelines are still missing, and radiologists and other clinicians disagree on what to report and how to manage the findings. Additionally, ethical and legal aspects should be considered when determining the approach to IFs.

We will explore the pros and cons of reporting IFs in LCS, focusing on key controversies.

### Screening for the ‘Big-3’

LC, chronic obstructive pulmonary disease (COPD), and cardiovascular disease (CVD), known as the “Big-3 diseases,” share common pathophysiological mechanisms and often coexist in similar risk groups. The early identification of these diseases could reduce their mortality [13]; notably, data from the National Lung Screening Trial (NLST) showed that about 10% of LC screenees died from respiratory causes other than LC [14].

The presence of pulmonary emphysema, detected in 24 to 63% of LCS participants, is independently associated with increased LC incidence and mortality, as well as higher all-cause and respiratory disease-related mortality [15, 16]. Reporting emphysema enables referral for clinical and functional assessment for those with moderate to severe lung involvement [17], and may support a risk-based approach, including shorter screening intervals and long-term screening duration for higher-risk subjects. Automated methods for detecting and quantifying emphysema in LCS are widely reported [18], though visual assessment remains an option [6]. A retrospective NLST analysis showed that a “rapid reading” of significant pulmonary abnormalities, like emphysema and fibrotic lung disease, aids in identifying individuals who later died of a respiratory cause [14].

Data from NLST and the Early Lung Cancer Action Project (ELCAP) demonstrated a positive correlation between CAC and CV-related mortality [19, 20]. European trials, including the NELSON, the Danish Lung Cancer Screening trial (DLCST), and the MILD trial [21–23], found that CAC scores > 400 are significantly associated with all-cause mortality. Despite some initial skepticism, CAC can be accurately detected and quantified on non-ECG gated LDCT scans, both visually and automatically [24]. Ascending thoracic aortic aneurysm, with a 3.5% prevalence, was the second most common vascular-related IF: appropriate management, including surveillance and cardiologist referral, may guide surgical decision-making [25].

Integrating quantitative CT data for COPD and CVD may enhance predictive models for LC, thus allowing for personalized screening intervals and cost reduction, by identifying those participants with lower LC risk who could be reevaluated at a longer interval [8]. Also, reporting emphysema findings could offer personalized motivation for tobacco cessation in current smokers and help primary care providers identify candidates to be referred for spirometric assessment, which might show evidence of airway obstruction—a key factor for diagnosing COPD—and to be potentially started on appropriate treatment. Despite interest in combining LC screening with secondary prevention of COPD and CVD, scientific evidence is awaited to further define its impact on morbidity or mortality, and precise cut-off values for referral remain to be assessed [26].

### Identification of extrapulmonary malignancies

**Fig. 1:**
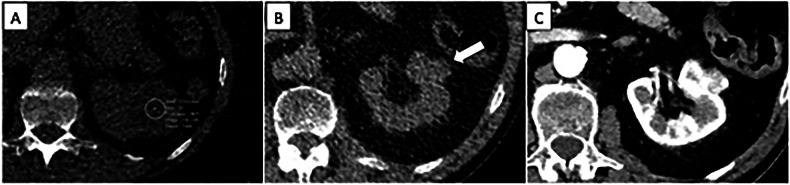
Challenges associated with the characterization of renal lesions. **A** Hypoattenuating lesion of the left kidney whose cystic or solid nature cannot be assessed due to a large deviation in attenuation measurement. **B** Exophytic lesion of the left kidney with high attenuation (white arrow); the extrathoracic IF was reported and evaluated by contrast-enhanced CT (**C**), which confirmed a lesion representing a stage I clear cell renal cell carcinoma. The detection of such a small exophytic renal lesion was due to a very attentive image review: in the setting of LCS, it can be debated if it should be considered a missed diagnosis if unmentioned

Based on NLST data, over 20% of the deaths in the LDCT arm were attributed to extrapulmonary cancers (including mediastinal, liver, pancreatic and kidney cancers), with renal and liver lesions being the most frequently reported significant IFs (Fig. 1) [11]. With the evaluation of subdiaphragmatic organs being only partial, chest LDCT cannot be considered a technique that provides a reliable assessment of extrathoracic organs. In the NLST, there were more individuals diagnosed with kidney, thyroid and liver cancers in the group without reported potentially significant findings [27]. Only a very small proportion of IFs are malignant: reported rates of extrapulmonary malignancies incidentally detected during LCS range from 0.05% [28] to 0.5% [27, 29, 30]. There is no scientific evidence on whether the early diagnosis of these malignancies will reduce mortality, morbidity and the economic burden associated with managing cancers at advanced stage [31].

### Screening of body composition and bone mineral density

Evidence links body composition to clinical and prognostic outcomes in LCS [32]. Xu et al demonstrated that automated measurement of body composition on baseline LDCT improved predictions for all-cause, LC-related and CVD-related mortality in the NLST [33]. LCS-LDCT may also offer valuable information on bone mineral density (BMD), a biomarker for osteoporosis, a condition responsible for an increased number of deaths and disability-adjusted life-years [34]. Deep learning techniques have shown high accuracy in measuring vertebral BMD for detecting osteoporosis and low BMD, representing a promising tool for automated opportunistic osteoporosis screening and could potentially reduce the need for additional tests like DEXA and their associated costs [35, 36]. The impact of body composition on mortality, morbidity, and cost-effectiveness within LCS is currently being investigated [37].

### Financial costs and impact on cost-effectiveness

LCS with LDCT can be cost-effective in high-risk individuals [38]. However, this often overlooks the financial burden of reporting and managing IFs. With as many as one in three to one in five LCS participants having potentially clinically significant IFs [11, 27], the impact on the healthcare cost might be substantial. Reporting IFs frequently results in additional diagnostic tests, follow-up imaging, and consultations with general practitioners (GPs) and specialists, potentially for benign or clinically insignificant findings. Management of IFs relies on their preliminary stratification into actionable and “not actionable” ones, with the former including those needing further assessment to clarify their nature. However, actionability represents a complex concept, only partly related to the underlying clinical significance. For instance, a hiatal hernia might not be inherently actionable, but it could explain the symptoms of the screenees [25]. Therefore, defining what is significant or actionable can be challenging and subject to the experience and confidence of the reading radiologists. There are European and American guidelines and recommendations available, but more scientific evidence based on data acquired in LDCT (and ULDCT) setting is still awaited [6, 39, 40].

To maintain cost-effectiveness, reporting significant IFs consistently and managing them according to guidelines is crucial [11]. A study by Morgan et al estimated that 46.2% of the calculated per-patient reimbursement associated with LCS was attributable to the evaluation and treatment of IFs [41]. Nonetheless, limited data shows that the costs to characterize clinically relevant IFs could be manageable. Based on NLST data, Gareen et al observed a little difference in total annual per-person costs between LDCT arm and chest X-ray arms despite a higher number of significant IFs detected in the LDCT arm [42]. In an Italian LCS trial, the average cost of radiologic follow-up studies for IFs at the baseline round was calculated to be $12.67 per participant [43]. Although their study was not intended to be a cost-effectiveness analysis, Bartlett et al observed that the overall costs due to incidental findings reporting was £5.69/participant in a UK LCS pilot [44].

Preliminary modeling studies suggest that “Big-3 screening” may be more cost-effective than LCS alone, largely due to the benefits derived from CVD screening, especially when individuals at high risk for CVD are included [45]. Further research is urgently needed to prove cost-effectiveness, which is essential for policymakers in designing screening strategies.

### Impact on radiologists and other resources in the healthcare system

Reporting IFs intrinsically increases radiologists’ workload for LDCT reporting and the potential additional imaging studies. Rampinelli et al found that ultrasound was the most frequently used diagnostic tool, performed in 81% of cases (245/303) [29]. Similarly, Priola et al reported that ultrasound of the upper abdomen and thyroid were the most common follow-up imaging studies [43]. These extra imaging tests add pressure to radiology departments already facing heavy workloads.

Standardized reporting systems can help ensure consistent evaluation and management of findings, potentially reducing the impact on radiologists’ workload; it is expected that radiologists include in their report adequate information on the following steps after the identification of actionable IFs [12]. Additionally, unchanged follow-up results could simplify and shorten the reporting, with two possible adoptable strategies: avoid reporting (as per NLST algorithm) or rapidly reporting an overall statement of stability. AI holds promise in enhancing the evaluation of IFs by recognizing IFs, potentially improving the accuracy of risk stratification and reducing unnecessary follow-up tests. A recent analysis of 24.401 baseline CT scans of the NLST reported that 3.880 displayed significant extrapulmonary IFs, with a fully automated AI model able to identify structures associated with high mortality risk [46]. Furthermore, deep learning software has shown promise in reducing interobserver variability for identifying interstitial lung abnormalities. However, integrating these systems into radiological workflows remains challenging [47].

A sub-study from an LCS pilot in West London examined the frequency and proportion of subsequent GP visits. In that cohort, 10.6% of participants (163/1542) were referred to primary care, with CV or respiratory issues accounting for 85.4% of referrals leading to changes in management in 38.1% and 12.7% of cases, respectively [44]. As LCS shifts from trials to population-based programs, the number of individuals undergoing screening is expected to rise significantly. If 10% of screened individuals require referrals, the burden on primary care will be considerable. Accurately assessing the true impact of IFs on healthcare workload remains remarkable, and workups should focus on IFs considered potentially significant [48, 49].

### Participants’ preferences and autonomy, and their psychological burden

LCS participants might experience short-term increased distress, which is not strictly related to detecting additional abnormalities but is inherent to the LCS pathway [50]. Effective communication with participants is crucial in managing all reported findings. Involving them in decision-making, respecting their values and preferences, and addressing their concerns can help alleviate anxiety and ensure alignment with their healthcare goals. The SUMMIT Study evaluated a strategy for sharing LDCT results using a booklet highlighting potential findings beyond pulmonary nodules. Participants reported that this approach improved communication and expressed a desire for more interaction with screening personnel, potentially boosting adherence to following recalls [51]. Sharing results can also create a teachable moment between the screenees and their GPs [52] and allows an easier understanding of the presence of these results in previous examinations [53]. On the other hand, Bartlett et al found that referral to GPs does not guarantee follow-up. Among 159 participants in a screening program who were found to have relevant IFs and agreed to follow-up (159/163), only 57.2% (91/159) attended their GP appointments. This means that more than 4 out of 10 participants did not follow up on the information indicating a health abnormality that required medical attention [44].

Overall, participants informed of the presence of significant IFs did not show a significant difference in health-related quality of life or state anxiety compared to those with negative results [11], supporting the perception of detecting significant IFs as a potential benefit of LCS [54, 55]. Moreover, to reduce the burden of reporting non-actionable IFs, a reasonable approach could be to report only those IFs that require further investigation; nonetheless, detecting actionable IFs could be challenging on low- or ultra-low-dose CT scanning protocols, and screenees should be fully informed of such limitation [6, 12]. The psychological impact of LCS is complex and influenced by multiple factors, including specific individual characteristics [50, 56]. Concrete data on the effects of reporting and referring IFs is still lacking.

### Overcalling and overdiagnosis risks

Overcalling refers to the over-interpretation of imaging results, where a radiologist identifies a finding as abnormal or significant when it is normal or insignificant. This can lead to false-positive results, unnecessary further testing, including invasive procedures and complications, anxiety, or even unnecessary treatments. Overdiagnosis occurs when conditions that are detected would not have caused symptoms or affected a person’s lifespan if left untreated. These are particularly relevant for IFs, as many might fall into these categories. Due to varying approaches in reporting and managing screen-detected IFs, the reported prevalence can vary widely. IFs are quite common, with rates reported as high as 94% and 2% to 13% of individuals requiring further evaluation [41, 57, 58]. However, most of these findings are benign and have no significant effect on morbidity or mortality. Quantifying the risk of overdiagnosis and overtreatment related to IFs is challenging, and reliable data on the subject are not yet available. To minimize these risks, the best approach is to be highly selective in reporting IFs, recognizing those that might be actionable or have clinical significance.

### Legal implications of recommendations regarding incidental findings

In cancer screening, avoiding detection errors is crucial, as they can delay management and allow the disease to progress to a more advanced stage. Litigation can influence medical practice, as has been reported for mammography screening in the US [59]. This risk can extend to LCS if guidelines require reporting every potentially malignant anomaly outside the chest, including organs in the upper abdomen that are only partially visible at the lung base. A retrospective study on incidental renal tumor detection in NLST participants found relatively low inter-reader agreement (0.47) for anomalies below the diaphragm, underlining the concept of challenging the evaluation of such abnormalities in LDCT studies [60]. Emphasizing this limitation is important to help protect radiologists from potential litigation if such tumors are missed.

### Future perspectives

In view of what has been discussed, there is a need for a balanced approach, where reporting is specifically focused on clinically significant and actionable findings, while clinically insignificant and non-actionable findings are excluded. This principle could serve as a valuable rule of thumb to avoid unnecessary resource burden. Advances in AI offer promising opportunities to streamline the detection and characterization of IFs [46, 61], potentially limiting the risk of overlooking clinically significant IFs [62]. However, further robust scientific evidence is still needed before implementing such systems into clinical practice. Further research is also required regarding screening for the Big-3, both from a scientific perspective as well as in terms of cost-effectiveness.

The ethical, legal and economic considerations and implications of incidental findings remain complex and can vary significantly across the diverse healthcare systems in European countries. Collaborating with National Advisory Committees on Bioethics can help ensure alignment with ethical standards and support the development of tailored implementation strategies that respect local healthcare contexts.

## Conclusion

IFs in LCS present both opportunities and challenges for screenees and healthcare professionals. Managing these findings requires a balanced approach guided by evidence-based guidelines, which are currently insufficient. Such guidance is essential to protect screenees from harm and radiologists from liability in case of overlooked or overdiagnosed IFs.

Reporting intrathoracic findings such as emphysema and CAC allows LCS to integrate secondary prevention strategies for COPD and CVD. This approach improves risk stratification of screenees in a cost-effective manner. Overall mortality studies, however, are still awaited to define the impact of reporting CAC, emphysema or the “Big 3.” For extrathoracic findings, emphasis should be placed on “actionable” ones. Since actionable IFs lack a strict definition, radiologists must use their clinical judgment to report what is essential.

## Abbreviations

BMD: Bone mineral density
CAC: Coronary artery calcification
COPD: Chronic obstructive pulmonary disease
CVD: Cardiovascular disease
LC: Lung cancer
LCS: Lung cancer screening
LDCT: Low-dose computed tomography
